# Imaging findings of basaloid squamous cell carcinoma: a case report

**DOI:** 10.1007/s11282-026-00951-5

**Published:** 2026-07-23

**Authors:** Varisa Assapattarapun, Hiroaki Shimamoto, Danielle Ayumi Nishimura, Naoko Takagawa, Noriko Yamao, Kosei Ueshima, Katsutoshi Hirose, Kaori Oya, Masashi Okumura, Akinori Takeshita, Kazuhide Matsunaga, Narikazu Uzawa, Sanjay M. Mallya, Fan-pei Gloria Yang

**Affiliations:** 1 Department of Oral and Maxillofacial Radiology, Graduate School of Dentistry, The University of Osaka, 1-8 Yamadaoka, Suita, 565-0871 Osaka Japan; 2 Department of Oral Surgery and Oral Medicine, Faculty of Dentistry, Srinakharinwirot University, 114 Sukhumvit 23, Watthana, Bangkok, 10110 Thailand; 3 Department of Oral and Maxillofacial Pathology, Graduate School of Dentistry, The University of Osaka, 1-8 Yamadaoka, Suita, 565-0871 Osaka Japan; 4 Department of Clinical laboratory, The University of Osaka Dental Hospital, 1-8 Yamadaoka, Suita, 565-0871 Osaka Japan; 5 Department of Oral & Maxillofacial Oncology and Surgery, Graduate School of Dentistry, The University of Osaka, 1-8 Yamadaoka, Suita, 565-0871 Osaka Japan; 6 Section of Oral and Maxillofacial Radiology, UCLA School of Dentistry, 10833 Le Conte Ave, Los Angeles, CA 90095-1668 USA; 7 Department of Foreign Languages and Literature, National Tsing Hua University, No.101, Section 2, Guangfu Rd., East District,, Hsinchu, 300013 Taiwan; 8 Center for Cognition and Mind Sciences, National Tsing Hua University, No.101, Section 2, Guangfu Rd., East District, Hsinchu, 300013 Taiwan

**Keywords:** Basaloid squamous cell carcinoma, Imaging findings, CT, MRI, PET-CT

## Abstract

Basaloid squamous cell carcinoma (BSCC) is a rare, high-grade variant of SCC. In the oral cavity, BSCC predominantly originates from the floor of the mouth and base of the tongue and is characterized by aggressive behavior, with frequent nodal and distant metastases. To date, no case has comprehensively reported BSCC findings across multiple imaging modalities in a single patient. A 78-year-old male presented with persistent erythema, swelling, and pain in the anterior mandible. Clinical examination revealed exposed bone with an exophytic soft tissue mass and cutaneous fistula. The lesion was initially suspected to represent osteoradionecrosis following prior management of SCC (pT1N0M0) with laser resection, neck dissection, and radiotherapy. However, disease recurrence could not be excluded. Panoramic radiography showed an ill-defined radiolucency in the anterior mandible. CT revealed a 55 × 20 × 48 mm inhomogeneously enhancing mass with associated bone destruction and extending to the skin. MRI demonstrated alveolar and labial cortical bone destruction, with soft tissue component isointense to muscle on T1-weighted images and heterogeneous high signal on T2-weighted and T1-weighted contrast enhanced images. PET-CT showed high FDG uptake (SUVmax: 17.2) without nodal or distant metastasis. Biopsy findings were suggestive of BSCC, which was confirmed by histopathologic examination of the resected lesion. This report highlights the utility of multimodal imaging in lesion characterization and supporting clinical decision-making, while the histopathological examination remains the gold standard for the definitive diagnosis. It also underscores the importance of vigilant follow-up in patients with oral BSCC for early detection of local recurrence and distant metastases.

## Introduction

Basaloid squamous cell carcinoma (BSCC) is a high-grade variant of squamous cell carcinoma (SCC) that was first described by Wain et al. in 1986 [1]. In 1991, the World Health Organization (WHO) recognized BSCC as a distinct entity in the classification of head and neck malignancies [2]. It is relatively rare and accounts for approximately 0.6% of oral SCCs [3]. BSCC primarily affects males older than 50 years. The most common sites of BSCC are the upper aerodigestive tract and, in the oral cavity, usually occurs at the base of the tongue, floor of the mouth and gingiva [4–6]. Clinically, BSCC often presents as an exophytic nodule with or without ulcers, firm to hard in consistency. The etiological risk factors of BSCC are similar to those of conventional SCC, including tobacco and alcohol consumption [7].

Compared with conventional oral SCC, oral BSCC tends to present a more advanced stage at the time of initial presentation, often with deep infiltration and lymph node and distant metastasis. Large, pooled analyses [4, 5] have confirmed its aggressive nature and propensity for metastasis and BSCC is consistently associated with a poor prognosis [1, 2, 7, 8]. It requires multimodality treatments including surgical resection and neck dissection, with or without post-surgical radiotherapy and/or chemotherapy.

Here, we report a case of BSCC arising in the anterior mandible and describe its characteristics on multiple imaging modalities. To our knowledge, no prior reports have comprehensively described multimodal imaging findings of BSCC in a single patient.

## Case report

A 78-year-old male was referred to the University of Osaka Dental Hospital due to persistent symptoms despite having undergone multiple previous treatments. Three and a half years prior to this visit, he underwent laser resection of a suspected granuloma on the floor of the mouth near the Wharton’s duct. Histopathologic evaluation identified the lesion as a moderately differentiated SCC (pT1N0M0). Several months later, the cancer had metastasized to a left upper jugular lymph node which was managed with neck dissection and postoperative radiotherapy (60 Gy). Ten months prior to presentation, the patient developed redness, swelling, and pain in the mandibular anterior region. The changes were attributed to osteoradionecrosis (ORN) and were managed with antibiotics and steroids. However, his condition remained unresponsive to the repeated treatments, including an incision and drainage.

**Fig. 1:**
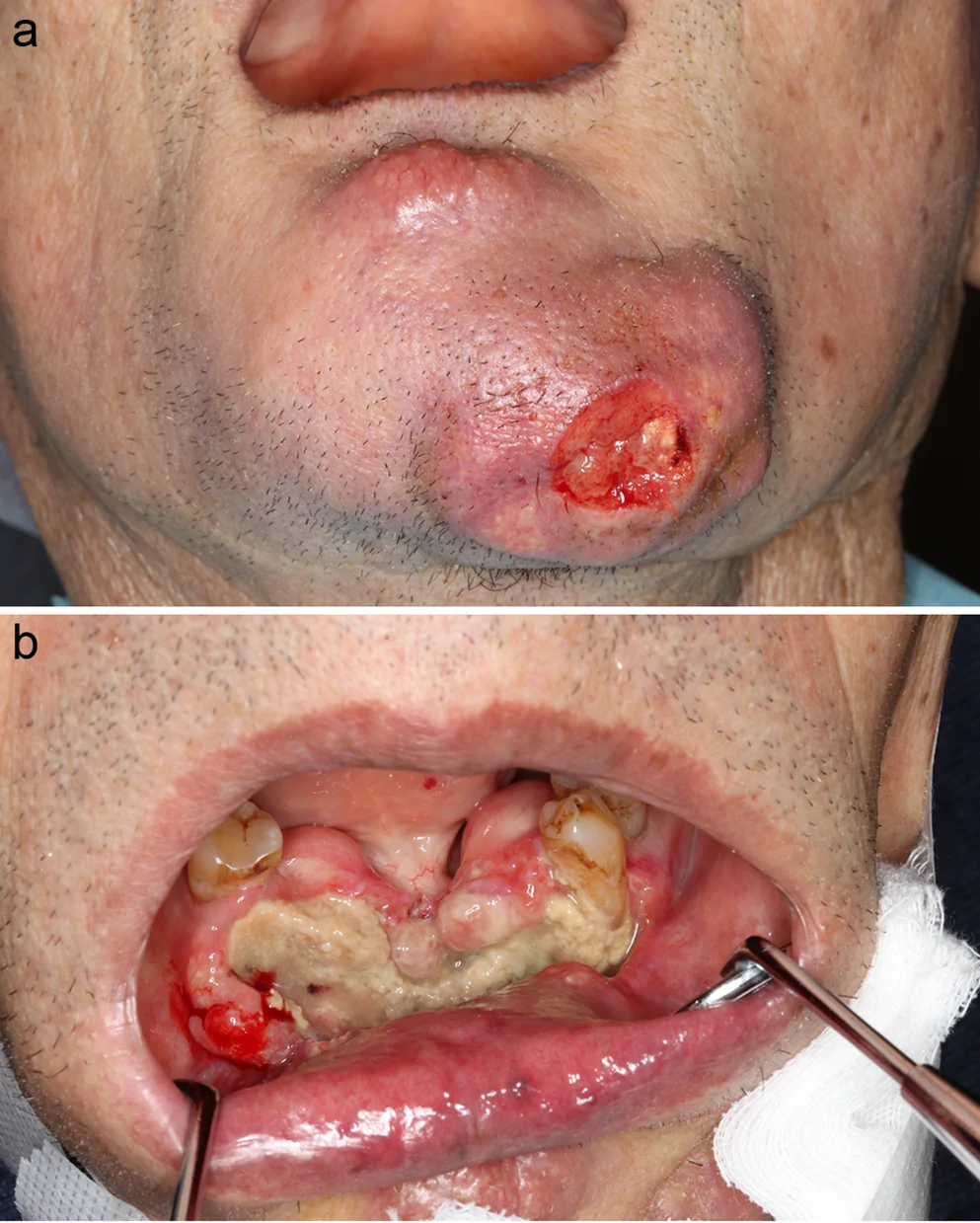
Clinical photographs. **a**. Extraoral photograph demonstrates an exophytic soft tissue mass in the mental region with erythema, swelling, and a cutaneous fistula. **b**. Intraoral photograph reveals exposed bone in the lower anterior edentulous region with an irregular overlying soft tissue surface

At the time of presentation to our clinic, an extraoral ulcerated soft tissue mass with redness, swelling and fistula was evident in the mental region (Fig. 1a). Intraoral examination revealed bony exposure in the mandibular anterior edentulous ridge with soft tissue ulceration and necrosis (Fig. 1b). The lesion was initially suspected to be osteoradionecrosis following prior radiotherapy, but the possibility of local tumor recurrence could not be ruled out.

**Fig. 2:**
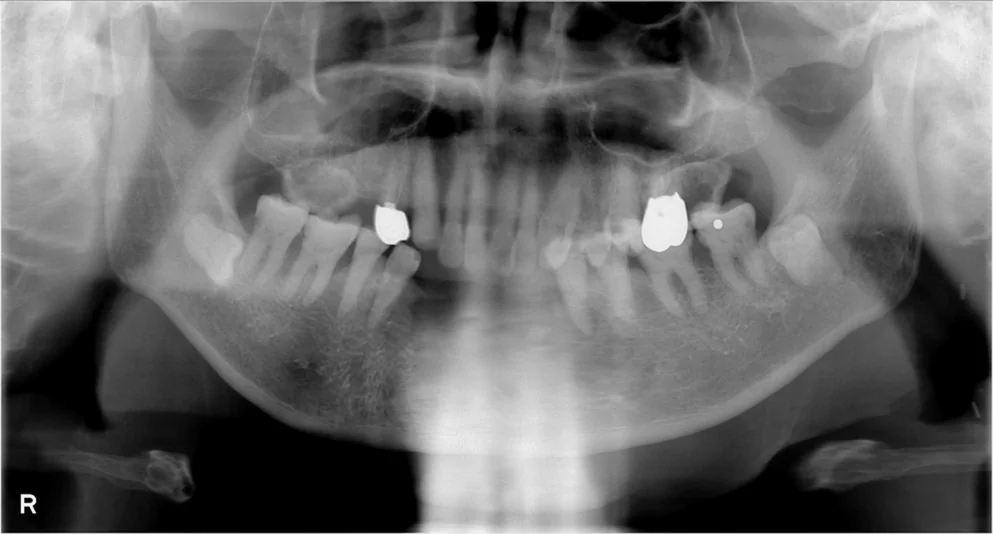
Panoramic radiograph shows an ill-defined radiolucent region at the anterior mandible

**Fig. 3:**
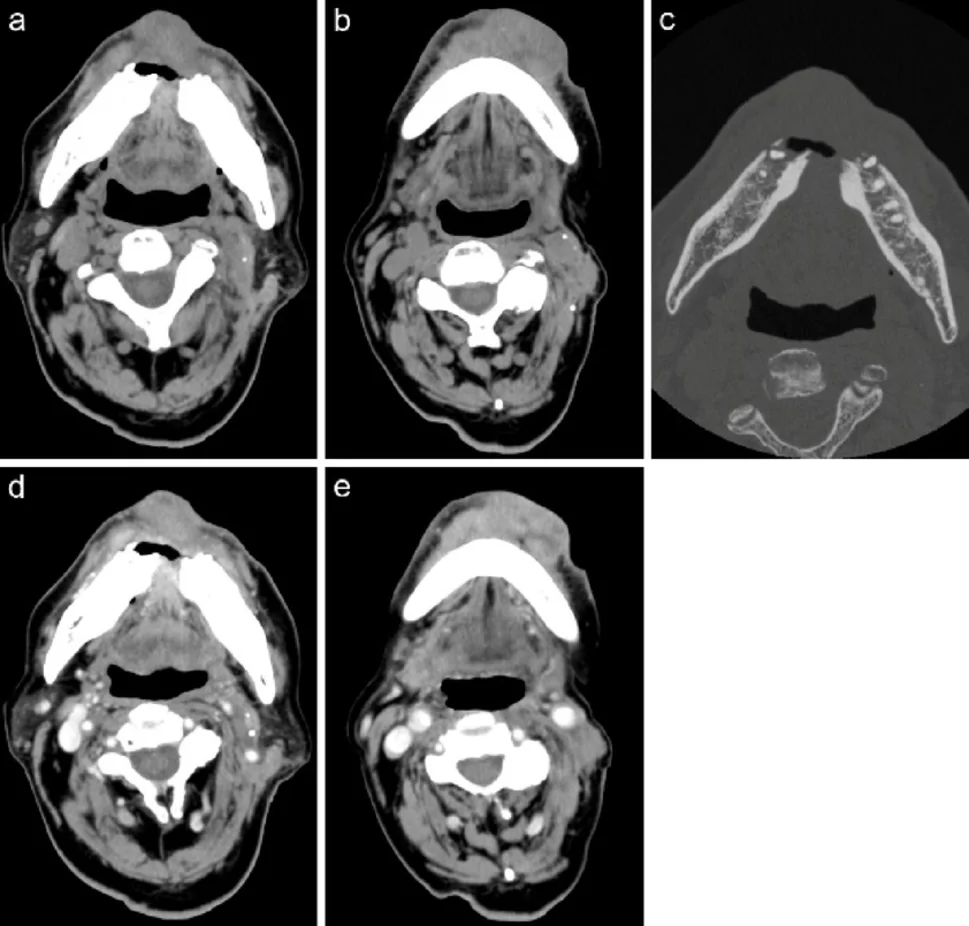
CT examination. **a**., **b**. Soft tissue window axial CT images demonstrate an ill-defined mass extending from the anterior mandible to the mental skin with associated bone destruction. **c**. Bone window axial CT image shows destruction of the anterior mandible. **d**., **e**. Contrast-enhanced axial CT image demonstrates inhomogeneous enhancement of the lesion with associated bone destruction

**Fig. 4:**
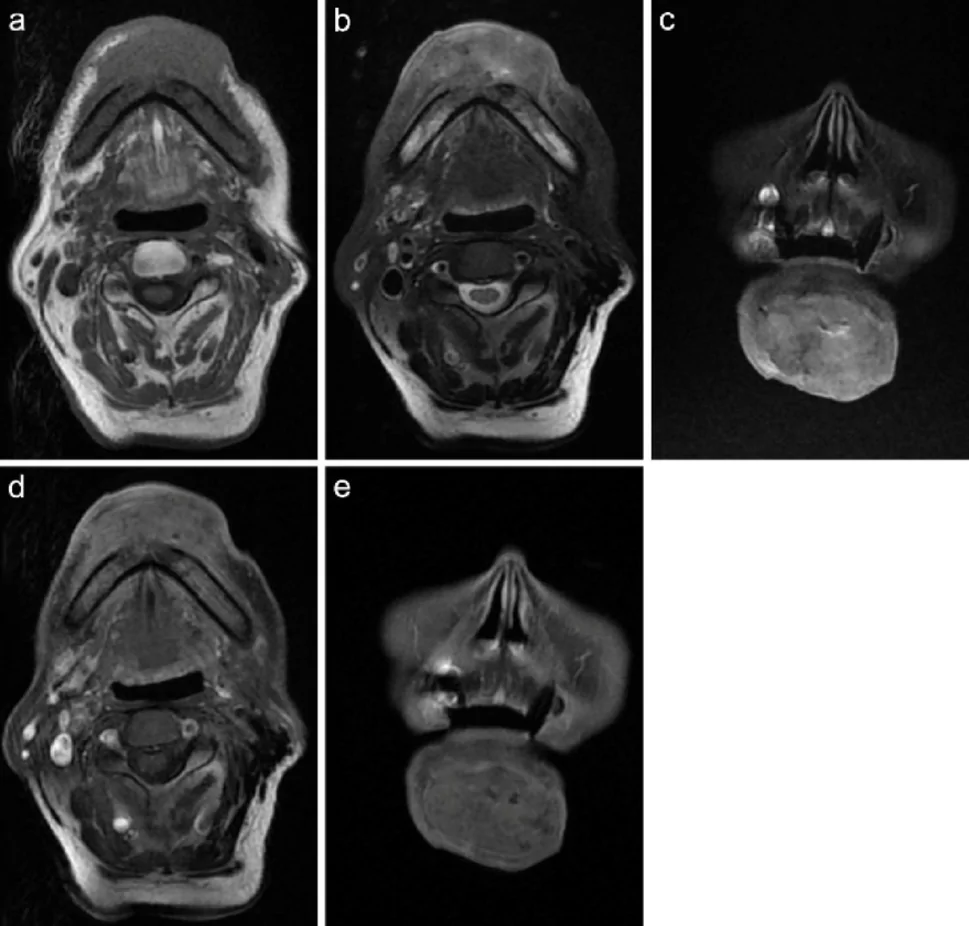
MRI examination. **a**. Axial T1-weighted image shows destruction of the alveolar bone and labial cortical plate of the anterior mandible. The associated soft tissue lesion is isointense to adjacent muscle. **b**., **c**. Axial and coronal T2-weighted images with fat suppression reveal heterogeneous high signal intensity. **d**., **e**. Axial and coronal gadolinium-enhanced T1-weighted images with fat suppression exhibit heterogeneous enhancement of the lesion

**Fig. 5:**
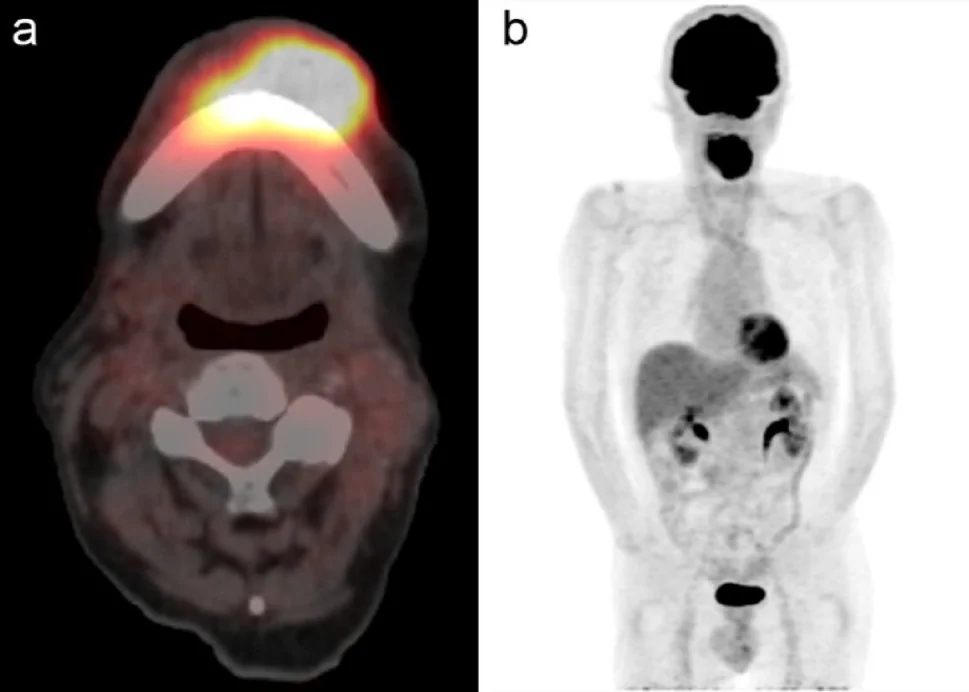
PET-CT examination. **a**. PET-CT image demonstrates high uptake of FDG in the anterior mandible and mental soft-tissue region with an SUVmax of 17.2. **b**. Maximum intensity projection image shows no evidence of lymph node metastasis and distant metastasis to other organs

Panoramic radiography showed an ill-defined radiolucency in the anterior mandible (Fig. 2). Computed tomography (CT) showed a 55 × 20 × 48 mm ill-defined mass extending from the anterior mandibular region to the mental skin surface with effacement of subcutaneous fat (Fig. 3a and b). Bone window image showed ill-defined bone destruction extending from the mandibular left first premolar to the mandibular right first premolar (Fig. 3c) with inhomogeneous contrast enhancement (Fig. 3d and e). Magnetic resonance imaging (MRI) revealed a 55 × 23 × 43 mm soft tissue mass isointense to muscle on T1-weighted images and with non-uniform high signal intensity on T2-weighted images and heterogeneous enhancement following gadolinium contrast administration. The mass caused destruction of the alveolar bone and labial cortical bone at the anterior mandible (Fig. 4). With diffusion-weighted imaging (DWI), the apparent diffusion coefficient (ADC) was low (0.83 × 10^− 3^ mm^2^/s). ^18^F-fluorodeoxyglucose (FDG) positron emission tomography (PET)-CT revealed a focal hypermetabolic area at the anterior mandibular region with a maximum standardized uptake value (SUVmax) of 17.2, which further supported the presence of a malignant neoplasm (Fig. 5a). There was no evidence of lymph node metastasis or distant metastasis to other organs (Fig. 5b).

**Fig. 6:**
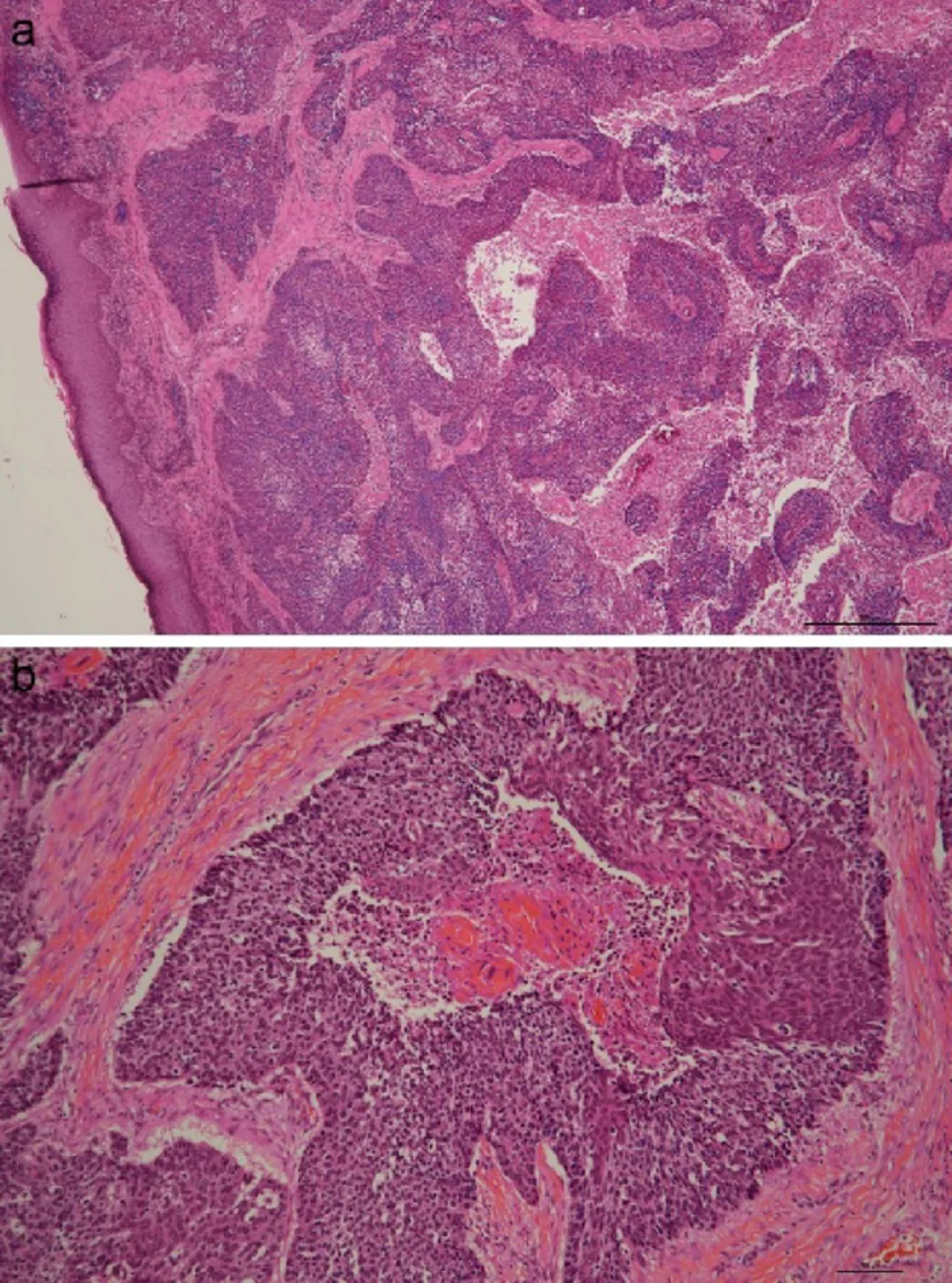
Histopathologic images. **a**. Low-power view of hematoxylin-eosin stained tissue sections demonstrates surface keratinized stratified squamous epithelium with invading nests of tumor cells in the underlying connective tissue (x4, bar = 500 μm). **b**. High magnification reveals the characteristic cellular features of basaloid squamous cell carcinoma, including pleomorphism, hyperchromatism, increased mitotic activity and high nucleus to cytoplasmic ratio of tumor cells. The tumor nests show central necrosis and peripheral palisade-like arrangement of basaloid cells (x10, bar = 100 μm)

An incisional biopsy was performed, and histopathologic evaluation identified the lesion as BSCC. The biopsy specimen was subjected to immunohistochemical analyses which demonstrated positive immunoreactivity to epithelial cell markers p63, CK5/6, and Ber-EP4 and was negative for reactivity to synaptophysin and chromogranin A. The patient underwent surgical resection and subsequent mandibular reconstruction. Histopathologic examination of the surgical specimen showed surface keratinized stratified squamous epithelium with invading nests of tumor cells in the underlying connective tissue (Fig. 6a). The tumor cells demonstrated pleomorphism, hyperchromatism, increased mitotic activity and a high nucleus-to-cytoplasmic ratio (Fig. 6b). Necrosis was present at the center of the tumor nests and a palisade-like arrangement of cells was present at the periphery of the nests. The final histopathologic diagnosis was BSCC (pT4a). Review of the histopathological slides originally diagnosed as moderately differentiated SCC at another institution demonstrated histological features consistent with those observed in our surgical specimen.

Lung metastases were detected on subsequent follow-up CT and PET-CT scans. The patient underwent a total of three partial lung resections at 34 months, 44 months and 47 months following surgical resection. Currently, the patient continues to undergo regular follow-up over 6 years postoperatively, with no evidence of tumor recurrence and metastasis except for ORN.

## Discussion

BSCC is a rare subgroup of SCC composed of basal cells and accounts for approximately 0.6% of oral squamous cell carcinoma [3]. The clinical presentations of BSCC are similar to those of conventional SCC. In the oral cavity, BSCC most commonly arises in the lower gingiva and the floor of the mouth [9]. Compared with conventional SCC, BSCC exhibits a higher frequency of both regional nodal involvement and distant metastasis, especially to the liver and the lungs. The rate of cervical lymph node metastasis ranges between 40% and 70% [10–13], whereas the frequency of distant metastasis can be as high as 75% [1, 7, 9, 13].

Basaloid squamous cell carcinoma has been consistently described as a deeply invasive tumor with submucosal spread and aggressive local infiltration, often presenting at an advanced T stage. The present case highlights the contributions of multi-modality diagnostic imaging of such lesions to characterize its local behavior and to identify regional and distant metastases for comprehensive treatment planning and rigorous post-operative monitoring of patients. Both CT and MRI provide valuable information on the soft-tissue extent of the lesion (Figs. 3 and 4), with MR providing superior definition of the local lesion infiltration into the soft-tissues (Fig. 4a-e). CT imaging provided better delineation of bone destruction (Fig. 3c). Notably, the characteristic features of central necrosis and internal cavitation observed in oral SCC are less frequent in BSCC [14]. In the present case, a central necrotic component was not identified on imaging. With DWI, the lesion had a low ADC, 0.83 × 10^− 3^ mm^2^/s, which is characteristic of malignancy, where high cellular density, high nuclear-to-cytoplasmic ratio, and reduced extracellular space restrict diffusion of water molecules. However, although the overall imaging features, including DWI, were suggestive of malignancy, definitive differentiation of oral BSCC from conventional oral SCC still relied on histopathological examination. The low ADC value observed in the present case appears to overlap with the reported ADC ranges of conventional oral SCC [15], emphasizing the limited specificity of DWI for distinguishing BSCC from conventional SCC.

On PET-CT, high FDG uptake (SUVmax: 17.2) demonstrated a high metabolic rate in the lesion, emphasizing the malignant nature and excluding distant metastases. Considering its high propensity for distant metastasis, Soriano et al. previously recommended that all BSCC patients undergo chest CT and FDG-PET for early detection of lung or other distant metastases [8]. Our report reinforces the potential role of FDG-PET in staging and detection of metastasis in BSCC, which may support clinical staging and treatment planning decisions. Overall, our comprehensive imaging approach facilitated accurate staging and appropriate management at various time points throughout the clinical course.

Histopathologically, BSCC typically consists of solid epithelial nests exhibiting a basaloid appearance and malignant cytologic features. Various growth patterns may be observed, including solid lobular, cribriform, cord-like, and trabecular nests, as well as glandular or cystic structures [5, 16]. The tumor cells are described as “basaloid” because of their immature morphology, resembling the basal layer cells of stratified squamous epithelium [17]. BSCC is frequently misdiagnosed as adenoid cystic carcinoma, basal cell adenocarcinoma, and neuroendocrine carcinoma. In the present case, the differential diagnoses of adenoid cystic carcinoma and basal cell adenocarcinoma were excluded based on the absence of distinct glandular structures and the strong expression of p63, CK5/6 and Ber-EP4 in the tumor cells. Neuroendocrine carcinoma was also ruled out, as the tumor cells were negative for the neuroendocrine markers synaptophysin and chromogranin A. Therefore, a final diagnosis of BSCC was made.

Currently, there are no standard treatment protocols for BSCC. Surgical resection remains the primary treatment, and is often combined with neck dissection for regional control. Adjuvant radiotherapy or chemoradiation is frequently recommended due to the high risk of recurrence and metastasis. In the present case, the management achieved satisfactory local control, underscoring the role of diagnostic imaging features in supporting treatment decisions with favorable outcomes.

When compared by stage, site and gender, the local recurrence rate of BSCC is similar to that of the conventional SCC. Although the recurrence rate is about 33% [13], the prognosis for BSCC is generally poor due to its aggressive nature and tendency to metastasize regionally and distantly. Reported 5-year overall survival rates range from 6% to 17.5% [8, 18, 19]. However, a study of BSCC in Japan revealed a markedly higher 5-year survival rate of 74.2%, attributed to the diagnosis of a high percentage (70.3%) of early-stage cases [9]. Even with early detection, accurate radiologic assessment of tumor extent and regional lymph node involvement is essential for determining the appropriate extent of surgical resection required to achieve tumor-free margins. Despite the high-grade nature of BSCC in the present case, including local recurrence, pulmonary metastases, and ORN, the patient remains under regular surveillance with no evidence of disease. This favorable outcome underscores the critical role of comprehensive radiologic evaluation in guiding treatment decisions, even in the setting of recurrent disease.

Among multimodal imaging, the radiological features in this case were non-specific and overlapped with those of conventional oral squamous cell carcinoma and post-radiotherapy changes such as osteoradionecrosis. Therefore, imaging alone is insufficient to establish a definitive diagnosis of BSCC. The primary value of imaging in this case lies more in lesion characterization, assessment of the lesion extent, identification of metastasis, and guidance of clinical management, rather than in providing disease-specific diagnostic features.

This report has inherent limitations because it is a single case report, which the findings cannot be generalized. The radiographic features observed, including DWI, are non-pathognomonic and demonstrate overlap with other malignancies. Therefore, these findings cannot be used to establish diagnostic criteria for oral BSCC. Further studies with larger case series or systematic reviews are needed to determine whether reproducible imaging features of BSCC exist.

## Conclusion

The favorable outcome in this case highlights the value of multimodal imaging in evaluating tumor extent and supporting treatment decisions. However, since imaging findings for BSCC are non-specific, histopathological confirmation is required. This case report emphasizes the importance of close follow-up in patients with oral BSCC for early detection of local recurrence and distant metastases.

## Data Availability

No datasets were generated or analysed during the current study.
